# Exploration of the Amazon

**Published:** 1854-04

**Authors:** 


### *Exploration of the Amazon.*

### HERNDON'S REPORT.

The above report, which was submitted to Congress the present Session, by Lieut. Herndon of the Navy, giving the particulars of his explorations of the river Amazon and some of its tributaries, is one of the most interesting documents which we have perused. It will be recollected that Lieut. Herndon was sent out by this Government, with the consent of the governments of Brazil and Peru, to examine this comparatively unknown region. The result has been most interesting and satisfactory in a commercial point of view, but what particularly interests us and has induced a notice of it in this Journal is the medical topography and the botany of this interesting region, gathered here and there from the report.

For the benefit of our medical readers who have not had an opportunity of perusing the report in detail, we will proceed to give such extracts as may prove interesting to our readers.

The expedition started from the city of Lima, Peru, on the 21st May, 1851, and, provided with all the necessary instuments for observation, passports, mules for transportation and muleteers, guides, &c., &c., the party were soon on their winding way to the Cordillera mountains. On the way he found the people residing in houses built of *adobe* or sun-dried bricks or cane and mud. The habits of the people are filthy,—domiciling with negroes, game cocks, and Guinea pigs. By pursuing the valleys, irrigated by streams with rapid currents, they found themselves rapidly rising the side of the mountains. Along these valleys the people were afflicted with the *tertiana* or chills and fevers. The only remedy which they use and of which they know anything, is spirits before the chill comes on, and during the fever, they use the juice of the bitter orange. When the case is very severe they send to Lima for medicine and advice.

By circuitous paths, rugged and steep acclivities, they finally reached the lowest line of the snow in which was buried the highest of the mountain peaks of that first range. He, in company with Lieut. Gibbon, who aided Herndon in this exploration, left the other members of the company and spurred their mules to the very apex of the mountain, amid snow, granite and dark porphyry rocks. Here was experienced by one of the party the consequences of breathing the rare atmosphere of these mountain elevations. But we will quote from the report.

"Gibbon, with the camera lucida, sketched the Cordillera. I expended a box of matches in boiling the snow for the atmospheric pressure; and poor Richards lay shivering on the ground, enveloped in our *pillons*, a martyr to the *veta*.

Veta is the sickness caused by the rarity of the atmosphere at these great elevations. The Indians called it veta, or vein, because they believe it is caused by veins of metal diffusing around a poisonous infection. It is a remarkable thing, that, although this affection must be caused by absence of atmospheric pressure, yet in no case except this, (and Richards was ill before,) that I have known or read of, was it felt at the greatest elevation, but always at a point below this—sometimes on one side, sometimes on the other. The affection displays itself in a violent headache, with the veins of the head swollen and turgid, a difficulty of respiration, and cold extremities. The smell of garlic is said to alleviate the symptoms; and the arrieros generally anoint their cattle over the eyes, and on the forehead, with an unguent made of tallow, garlic, and wild marjoram, as a preventive, before attempting the ascent. I did not observe that our animals were affected, though they trembled and breathed hard, which, I think, was attributable to the steepness of the hill up which we rode. The barometer stood at 16.730, indicating an elevation of sixteen thousand and forty-four feet. Water boiled at 182°.5; temperature of the air, 43°."

Their movement through the mountains was slow, owing partly to the delay necessary in examining the silver mines of this region, and partly on account of the difficulties which opposed themselves to their progress. He suffered here from an affection which arose either from the labor and toils consequent upon such explorations, or some electric condition. He says :—

"*July* 9.—Suffering from an affection called *macolca*, which is incident to nearly every one on his first visit to the mines. This is a painful soreness of the muscles, particularly on the front of the thigh. I could scarcely bear that my legs should be touched, and locomotion was anything but agreeable."

They arrived at the town of Cerro Pasco, which is thirteen thousand, and upward, feet above the level of the sea. It is a mining town, and contains from six to fifteen thousand souls. The population is, like every other community collected in mining districts, governed by avarice and the lust for money, and where the feelings of the heart are suppressed and the ordinary amenities of life contemned. He describes the climate as most disagreeable:—

"The temperature is so rigorous here that the hens do not hatch, nor the llamas procreate; and women, at the period of their confinement, are obliged to seek a more genial climate, or their offspring will not live.

Persons recently arrived, particularly if they have weak lungs, suffer from affections of the chest and difficulty of breathing. The miners suffer paralysis from the sudden changes of temperature to which they are exposed in and out of the mines, and from inhaling the fumes of the mercury in the operation of distilling. Those who suffer in this way are called *azogados*, from *azogue*, (quicksilver.) The most common diseases are pleurisies, rheumatisms, and a putrid fever called *tabardillo*. Pleurisies are said to be cured by taking an infusion of *mullaca*, an herb which grows in the neighborhood. It has very small leaves, and gives a small, round, red fruit."

They arrived at the city of Hannuco, one of the most ancient cities of Peru, and situated on the banks of the Hanllaga river. The climate here is more favorable than the last named city. Of this he says :—

"The climate of Huanuco is very equable and very salubrious.—There are no cases of affection of the chest which commence here; on the contrary, people with diseases caused by the inclemency of the weather about Cerro Pasco, come to Huanuco to be cured. Dysentery and tabardillo are the commonest diseases; and I see many people (particularly women) with goitre. I saw a women who had one that seemed to arise under each ear and encircle the throat like an inflated life-preserver. The affection is said to be owing to the impurity of the water, which is not fit to drink unless filtered. The lower class of people do not attend to this, and thus the disease is more general with them than with the higher classes."

About the 1st of August the explorer arrived at Tingo Marie, just below the junction of the Huallago with the river Monzon. It is proper to state here that previous to this, the party divided, one to proceed under Herndon himself, and the other under the direction of Lieut. Gibbon,—the former to reach the head-waters of the principal tributaries of the Amazon, the other by the tributaries of the Madeira, which empties into the Amazon low down, and to pursue that stream to its mouth. From Lima to Tingo Marie, it is 335 miles, the latter being an Indian village of one hundred and eighty-eight population and of the tribe of Cholones. Of the diseases we extract the following:—

"The common diseases are lymphatic swellings of the body and limbs, (supposed to be caused by exposure to the great humidity of the atmosphere while fishing at night,) and *sarna*, (a cutaneous affection, which covers the body with sores, making the patient a loathsome object.) These sores dry up and come off in scabs, leaving blotches on the skin, so that an Indian is frequently seen quite mottled. I imagine it is caused by want of cleanliness, and the bites of the sand-flies. They take, as a remedy, the dried root of a small tree called *sarnango*, grated and mixed with water. It is said to have a powerfully-intoxicating and stupefying effect, and to cause the skin to peel off."

An instance is given in the extract below of the capacity for improvement of the special senses. The extract also refers to a peculiar physical conformation of that particular class of monkeys, of rare interest to the comparative anatomist.

"These Indians have very keen senses, and see and hear things that are inaudible and invisible to us. Our canoe-men this morning commenced paddling with great vigor. I asked the cause, and they said that they heard monkeys ahead. I think we must have paddled a mile before I heard the sound they spoke of. When we came up to them, we found a gang of large red monkeys in some tall trees on the river-side, making a noise like the grunting of a herd of enraged hogs. We landed, and in a few minutes I found myself beating my way through the thick undergrowth, and hunting monkeys with as much excitement as I had ever hunted squirrels when a boy. I had no balls with me, and my No. 3 shot only served to sting them from their elevated position in the tops of the trees, and bring them within reach of the pucunas of the Indians. They got two and I one, after firing about a dozen shot into him. I never saw animals so tenacious of life; this one was, as the Indians expressed it, *bathed in shot* (*banado en municion.*) These monkeys were about the size of a common terrier-dog, and were clad with a long, soft, maroon-colored hair; they are called *cotomonos*, from a large goitre (coto) under the jaw. This is an apparatus of thin bone in the wind-pipe, by means of which they make their peculiar noise. The male, called *curaca*, (which is also the appellation of the chief of a tribe of Indians,) has a long red beard. They are called *guariba* in Brazil, where they are said to be black as well as red; and I believe they are of the species commonly called *howling monkeys*.

It is scarcely worth while to say that the Indians use parts of this animal for the cure of diseases, for I know no substance that could possibly be used as a remedial agent that they do not use for that purpose. The mother carries the young upon her back until it is able to go alone. If the dam dies, the sire takes charge. There are vast numbers in all the course of the river, and no day passes to the traveller that they are not heard or seen."

The above extracts will be sufficient for the present. We shall again draw on the report for some further interesting facts for future numbers. We regret that the expedition was not complete. The numerous interesting facts to the medical man, although elicited by one unacquainted with medicine, show clearly that much more might have been obtained, had the explorer been accompanied by a few scientific medical men. The specimens of comparative anatomy, of medical and economical botany, and the diseases found in that valley, with their pathological characteristics and their causes, would be subjects of the first consideration and importance. It is very apparent from this report that there is no region from which we have heard, or of which we know anything, promising so much for the scientific explorer, and we regret very much that Government did not complete the work, and trust that it will yet obtain permission, from the governments through whose territory this mighty river flows, for a scientific examination, and that a corps be immediately organized and despatched for that purpose. M'.

